# Supplementary material for: Multi-omic analysis of stroke recurrence in African Americans from the Vitamin Intervention for Stroke Prevention (VISP) clinical trial
Source: PLoS One. 2021 Mar 4;16(3):e0247257. doi: 10.1371/journal.pone.0247257 (PMC7932724; doi:10.1371/journal.pone.0247257)
Supplement: S1 Table — (DOCX) [file pone.0247257.s002.docx]

**S1 Table. Baseline demographics of matched pairs.**

|  | **VISP**  **Nonrecurrent Stroke**  **(N=22)** | **VISP**  **Recurrent**  **Stroke**  **(N=22)** | **P** | **Absolute SMD** |
| --- | --- | --- | --- | --- |
| **Age,** yrs^a^ | 63.68 (9.76) | 63.45 (11.83) | 0.945 | 0.021 |
| **Sex**  Males  Females | 11 (50.0%)  11 (50.0%) | 11 (50.0%)  11 (50.0%) | 1.000 | <0.001 |
| **RSS**  0  1  2  3 | 0 (0.0%)  14 (63.6%)  7 (31.8%)  1 (4.5%) | 1 (4.5%)  13 (59.1%)  0 (0.0%)  8 (36.4%) | 0.004 | 1.329 |
| **Cigarettes per day** | 1.50 (3.67) | 1.91 (4.88) | 0.755 | 0.095 |
| ^a^ Continuous traits described as mean (SD). Categorical traits described as N (%).  **Abbreviations**: RSS-modified Rankin Stroke Scale; SMD: absolute standardized mean difference ((mean 1- mean 2)/pooled SD) | | | | |
