# Supplementary material for: Multi-omic analysis of stroke recurrence in African Americans from the Vitamin Intervention for Stroke Prevention (VISP) clinical trial
Source: PLoS One. 2021 Mar 4;16(3):e0247257. doi: 10.1371/journal.pone.0247257 (PMC7932724; doi:10.1371/journal.pone.0247257)
Supplement: S2 Table — (DOCX) [file pone.0247257.s003.docx]

**S2 Table. Demographics by pair.**

|  | **VISP Recurrent Stroke** | | | | | **VISP Nonrecurrent Stroke** | | | |
| --- | --- | --- | --- | --- | --- | --- | --- | --- | --- |
|  | **Age** | **Sex** | **RSS** | **Cigs/day** | **Age** | | **Sex** | **RSS** | **Cigs/day** |
| Pair 1 | 42 | Male | 3 | 20 | 48 | | Male | 2 | 15 |
| Pair 2 | 42 | Male | 1 | 4 | 50 | | Male | 1 | 6 |
| Pair 3 | 53 | Male | 0 | 0 | 51 | | Male | 1 | 0 |
| Pair 4 | 60 | Male | 1 | 10 | 59 | | Male | 1 | 6 |
| Pair 5 | 62 | Male | 3 | 0 | 68 | | Male | 2 | 0 |
| Pair 6 | 62 | Male | 3 | 0 | 69 | | Male | 1 | 0 |
| Pair 7 | 72 | Male | 1 | 0 | 72 | | Male | 1 | 0 |
| Pair 8 | 73 | Male | 1 | 0 | 74 | | Male | 1 | 0 |
| Pair 9 | 76 | Male | 1 | 0 | 76 | | Male | 1 | 0 |
| Pair 10 | 77 | Male | 1 | 0 | 71 | | Male | 2 | 0 |
| Pair 11 | 81 | Male | 1 | 0 | 81 | | Male | 1 | 0 |
| Pair 12 | 46 | Female | 1 | 0 | 50 | | Female | 1 | 0 |
| Pair 13 | 53 | Female | 3 | 0 | 54 | | Female | 2 | 0 |
| Pair 14 | 55 | Female | 1 | 0 | 56 | | Female | 1 | 0 |
| Pair 15 | 58 | Female | 3 | 0 | 58 | | Female | 1 | 0 |
| Pair 16 | 59 | Female | 3 | 0 | 59 | | Female | 1 | 0 |
| Pair 17 | 62 | Female | 1 | 0 | 64 | | Female | 1 | 0 |
| Pair 18 | 65 | Female | 1 | 8 | 62 | | Female | 2 | 6 |
| Pair 19 | 70 | Female | 1 | 0 | 64 | | Female | 2 | 0 |
| Pair 20 | 72 | Female | 3 | 0 | 65 | | Female | 3 | 0 |
| Pair 21 | 77 | Female | 3 | 0 | 74 | | Female | 2 | 0 |
| Pair 22 | 79 | Female | 1 | 0 | 76 | | Female | 1 | 0 |
| **Abbreviations:** RSS- Rankin Stroke Scale; Cigs/day- cigarettes smoked per day | | | | | | | | | |
