# Supplementary material for: Multi-omic analysis of stroke recurrence in African Americans from the Vitamin Intervention for Stroke Prevention (VISP) clinical trial
Source: PLoS One. 2021 Mar 4;16(3):e0247257. doi: 10.1371/journal.pone.0247257 (PMC7932724; doi:10.1371/journal.pone.0247257)
Supplement: S3 Table — Gene region based on hg19. (DOCX) [file pone.0247257.s004.docx]

**S3 Table. Sphingolipid related genes used in SNP-based tests.** Gene region based on hg19.

| **Gene** | **CHR** | **Gene region (±10kb)** |
| --- | --- | --- |
| *CERS2* | 1 | 149194273-149224103 |
| *DEGS1* | 1 | 222427533-222457765 |
| *CERS6* | 2 | 169011005-169349890 |
| *CERS6-AS1* | 2 | 169326707-169361185 |
| *SGPP2* | 2 | 222987566-223141861 |
| *SGMS2* | 4 | 109023879-109061558 |
| *ASAH1* | 8 | 17948205-17996159 |
| *NSMAF* | 8 | 59666313-59744940 |
| *ACER2* | 9 | 19388925-19452500 |
| *SPTLC1* | 9 | 93823248-93927511 |
| *ASAH2* | 10 | 51607078-51688319 |
| *SGMS1* | 10 | 51725351-52063743 |
| *ASAH2B* | 10 | 52159714-52194575 |
| *SMPD1* | 11 | 6358231-6382584 |
| *ACER3* | 11 | 76239565-76422498 |
| *CERS5* | 12 | 48799356-48857583 |
| *SGPP1* | 14 | 63210688-63274509 |
| *CERS3* | 15 | 98748123-98912448 |
| *SPHK1* | 17 | 71874337-71905536 |
| *ACER1* | 19 | 6247725-6294562 |
| *CERS4* | 19 | 8170254-8243304 |
| *CERS1* | 19 | 18830355-18877953 |
| *SPHK2* | 19 | 53804360-53835474 |
| *CERK* | 22 | 45448971-45496854 |
| **Abbreviations**: CHR- chromosome; kb-kilobase pairs; *CERS2* -ceramide synthase 2; *DEGS1*- delta(4)-desaturase, sphingolipid 1; *CERS6*- ceramide synthase 6; *CERS1-AS1*- ceramide synthase 6 antisense RNA 1; *SGPP2*- sphingosine-1-phosphate phosphatase 2; *SGMS2*- sphingomyelin synthase 2; *ASAH1*- N-acylsphingosine amidohydrolase 1; *NSMAF*- Neutral sphingomyelinase activation associated factor; *ACER2-* alkaline ceramidase 2; *SPTLC1-* serine palmitoyltransferase long chain base subunit 1; *ASAH2*- N-acylsphingosine amidohydrolase 2; *SGMS1-* sphingomyelin synthase 1*; ASAH2B-* N-acylsphingosine amidohydrolase 2B; *SMPD1-* sphingomyelin phosphodiesterase 1; *ACER3-*  alkaline ceramidase 3; *CERS5*- ceramide synthase 5; *SGPP1-* sphingosine-1-phosphate phosphatase 1; *CERS3-* ceramide synthase 3; *SPHK1-* sphingosine kinase 1; *ACER1-* alkaline ceramidase 1;  *CERS4-* ceramide synthase 4; *CERS1-* ceramide synthase 1;  *SPHK2-* sphingosine kinase 2;  *CERK-* ceramide kinase | | |
