# Supplementary material for: Multi-omic analysis of stroke recurrence in African Americans from the Vitamin Intervention for Stroke Prevention (VISP) clinical trial
Source: PLoS One. 2021 Mar 4;16(3):e0247257. doi: 10.1371/journal.pone.0247257 (PMC7932724; doi:10.1371/journal.pone.0247257)
Supplement: S4 Table — (DOCX) [file pone.0247257.s005.docx]

**S4 Table. Summary of Welch’s *t-*test comparisons.**

| **Welch’s *t­-*test comparisons** | **Group 1 (n)** | **Group 2 (n)** |
| --- | --- | --- |
| VISP recurrent stroke | Yes (28) | No (22) |
| Recurrent stroke ever | Yes (12) | No (38) |
| Composite vascular endpoint | Yes 31) | No (19) |
| Treatment arm | High dose (23) | Low dose (27) |
| Baseline diabetes status | Yes (20) | No (30) |
| Current (at trial enrollment) smoker | Yes (11) | No (39) |
| Smoker ever | Yes (29) | No (21) |
| Male VISP recurrent stroke | Yes (16) | No (11) |
| Female VISP recurrent stroke | Yes (12) | No (11) |
| High dose VISP recurrent stroke | Yes (13) | No (10) |
| Low dose VISP recurrent stroke | Yes (15) | No (12) |
| Current smoker-yes, VISP recurrent stroke | Yes (7) | No (4) |
| Current smoker-no, VISP recurrent stroke | Yes (21) | No (18) |
| Smoker ever, VISP recurrent stroke | Yes (18) | No (11) |
| Never smoker, VISP recurrent stroke | Yes (10) | No (11) |
| Baseline diabetes-yes, VISP recurrent stroke | Yes (8) | No (12) |
| Baseline diabetes-no, VISP recurrent stroke | Yes (20) | No (10) |
