# Supplementary material for: Multi-omic analysis of stroke recurrence in African Americans from the Vitamin Intervention for Stroke Prevention (VISP) clinical trial
Source: PLoS One. 2021 Mar 4;16(3):e0247257. doi: 10.1371/journal.pone.0247257 (PMC7932724; doi:10.1371/journal.pone.0247257)
Supplement: S5 Table — (DOCX) [file pone.0247257.s006.docx]

**S5 Table. One-sample *t*-test of the fold change from 22 matched pairs.**

| **Metabolite** | **Subpathway** | **Nonrecurrent Mean** | **Recurrent Mean** | **Fold Change** | **P^a^** |
| --- | --- | --- | --- | --- | --- |
| gamma-glutamylhistidine | Gamma-glutamyl Amino Acid | 1.45 | 0.87 | 0.60 | 3.10E-04 |
| sphingomyelin (d18:1/20:1) | Sphingomyelins | 1.21 | 0.93 | 0.77 | 3.50E-03 |
| sphingomyelin (d18:2/21:0) | Sphingomyelins | 1.29 | 0.85 | 0.66 | 4.21E-03 |
| tricosanoyl sphingomyelin (d18:1/23:0) | Sphingomyelins | 1.25 | 0.90 | 0.72 | 7.16E-03 |
| iminodiacetate (IDA) | Chemical | 0.92 | 1.17 | 1.27 | 8.14E-03 |
| sphingomyelin (d18:1/20:0) | Sphingomyelins | 1.12 | 0.87 | 0.78 | 8.49E-03 |
| sphingomyelin (d18:2/18:1) | Sphingomyelins | 1.42 | 0.91 | 0.64 | 8.74E-03 |
| sphingomyelin (d18:2/23:0) | Sphingomyelins | 1.23 | 0.91 | 0.74 | 9.55E-03 |
| sphingomyelin (d18:1/19:0) | Sphingomyelins | 1.23 | 0.93 | 0.72 | 1.08E-02 |
| thioproline | Chemical | 0.94 | 1.16 | 1.23 | 1.22E-02 |
| 1-oleoyl-2-docosahexaenoyl-GPC (18:1/22:6) | Phosphatidylcholine (PC) | 1.16 | 0.91 | 0.78 | 1.25E-02 |
| lignoceroyl sphingomyelin (d18:1/24:0) | Sphingomyelins | 1.18 | 0.89 | 0.75 | 1.44E-02 |
| gamma-glutamylalanine | Gamma-glutamyl Amino Acid | 1.41 | 0.84 | 0.591 | 1.49E-02 |
| behenoyl sphingomyelin (d18:1/22:0) | Sphingomyelins | 1.14 | 0.86 | 0.76 | 1.53E-02 |
| gamma-glutamyl-alpha-lysine | Gamma-glutamyl Amino Acid | 1.22 | 0.87 | 0.71 | 1.54E-02 |
| sphingomyelin (d18:1/22:1) | Sphingomyelins | 1.17 | 0.93 | 0.79 | 1.57E-02 |
| salicyluric glucuronide | Drug - Analgesics, Anesthetics | 1.25 | 0.57 | 0.45 | 1.65E-02 |
| cis-urocanate | Histidine Metabolism | 1.23 | 0.76 | 0.62 | 1.67E-02 |
| gamma-glutamylthreonine | Gamma-glutamyl Amino Acid | 1.27 | 0.95 | 0.75 | 1.70E-02 |
| sphingomyelin (d17:2/16:0) | Sphingomyelins | 1.35 | 0.96 | 0.71 | 1.91E-02 |
| 4-allylphenol sulfate | Food Component/Plant | 2.69 | 1.11 | 0.41 | 2.01E-02 |
| sphingomyelin (d18:1/24:1) | Sphingomyelins | 1.17 | 0.94 | 0.81 | 2.08E-02 |
| trimethylamine N-oxide | Phospholipid Metabolism | 1.53 | 0.96 | 0.62 | 2.12E-02 |
| sphingomyelin (d18:1/21:0) | Sphingomyelins | 1.23 | 0.91 | 0.74 | 2.20E-02 |
| gamma-glutamylglutamate | Gamma-glutamyl Amino Acid | 1.50 | 0.94 | 0.63 | 2.30E-02 |
| taurine | Methionine, Cysteine, SAM and Taurine Metabolism | 1.11 | 0.87 | 0.78 | 2.37E-02 |
| 5-(galactosylhydroxy)-L-lysine | Lysine Metabolism | 1.60 | 0.96 | 0.60 | 2.42E-02 |
| sphingomyelin (d18:2/23:1) | Sphingomyelins | 1.24 | 0.96 | 0.78 | 2.43E-02 |
| N-acetylneuraminate | Aminosugar Metabolism | 1.13 | 0.90 | 0.80 | 2.45E-02 |
| indoleacetate | Tryptophan Metabolism | 0.88 | 1.66 | 1.89 | 2.51E-02 |
| gamma-glutamylglycine | Gamma-glutamyl Amino Acid | 1.35 | 0.82 | 0.60 | 2.72E-02 |
| sphingomyelin (d18:1/17:0) | Sphingomyelins | 1.25 | 0.96 | 0.77 | 2.81E-02 |
| sphingomyelin (d18:2/16:0) | Sphingomyelins | 1.30 | 1.01 | 0.78 | 2.97E-02 |
| creatinine | Creatine Metabolism | 1.14 | 0.98 | 0.86 | 3.01E-02 |
| phenylpyruvate | Phenylalanine Metabolism | 0.95 | 1.21 | 1.27 | 3.22E-02 |
| EDTA | Chemical | 0.99 | 1.30 | 1.32 | 3.23E-02 |
| methylsuccinate | Leucine, Isoleucine and Valine Metabolism | 1.31 | 0.99 | 0.76 | 3.40E-02 |
| octadecenedioate (C18:1-DC) | Fatty Acid, Dicarboxylate | 0.89 | 1.37 | 1.53 | 3.59E-02 |
| oleoyl ethanolamide | Endocannabinoid | 1.36 | 0.96 | 0.70 | 3.61E-02 |
| histidine betaine (hercynine) | Food Component/Plant | 1.32 | 0.74 | 0.56 | 3.81E-02 |
| 3-carboxy-4-methyl-5-pentyl-2-furanpropionate (3-CMPFP) | Fatty Acid, Dicarboxylate | 1.22 | 0.91 | 0.75 | 3.83E-02 |
| 5-acetylamino-6-formylamino-3-methyluracil | Xanthine Metabolism | 1.28 | 0.62 | 0.49 | 4.18E-02 |
| dihydroferulic acid sulfate | Food Component/Plant | 1.07 | 0.27 | 0.25 | 4.20E-02 |
| suberate (C8-DC) | Fatty Acid, Dicarboxylate | 1.24 | 0.94 | 0.76 | 4.20E-02 |
| N1-methylinosine | Purine Metabolism, (Hypo)Xanthine/Inosine containing | 1.34 | 1.05 | 0.78 | 4.27E-02 |
| ceramide (d18:1/20:0) | Ceramides | 1.14 | 0.97 | 0.85 | 4.50E-02 |
| alpha-hydroxycaproate | Fatty Acid, Monohydroxy | 0.75 | 1.03 | 1.37 | 4.61E-02 |
| sphingomyelin (d18:1/25:0) | Sphingomyelins | 1.20 | 0.93 | 0.77 | 4.78E-02 |
| sphingomyelin (d17:1/14:0) | Sphingomyelins | 1.16 | 0.91 | 0.78 | 4.83E-02 |
| ^a^ Statistical significance threshold p≤5.42e-05; suggestive threshold p≤2.27e-03 | | | | | |
