# Supplementary material for: Multi-omic analysis of stroke recurrence in African Americans from the Vitamin Intervention for Stroke Prevention (VISP) clinical trial
Source: PLoS One. 2021 Mar 4;16(3):e0247257. doi: 10.1371/journal.pone.0247257 (PMC7932724; doi:10.1371/journal.pone.0247257)
Supplement: S6 Table — (DOCX) [file pone.0247257.s007.docx]

**S6 Table. WGCNA module-trait associations for methylation profiles and stroke traits/outcomes.**

| **Module** | **# of Loci/Module** | **Correlated Trait** | **r** | **Pvalue^a^** |
| --- | --- | --- | --- | --- |
| thistle4 | 173 | PNS | 0.91 | 2.00e-18 |
| lightsteelblue1 | 214 | PNS | -0.89 | 6.00e-17 |
| tan4 | 172 | F12 | 0.88 | 2.00e-16 |
| indianred4 | 130 | Sex | 0.86 | 2.00e-14 |
| deeppink | 180 | Sex | -0.84 | 8.00e-14 |
| thistle1 | 165 | Folate | -0.83 | 4.00e-13 |
| palevioletred3 | 378 | F12 | -0.81 | 4.00e-12 |
| mediumorchid | 284 | TM | -0.78 | 1.00e-10 |
| darkolivegreen | 284 | Folate | 0.75 | 2.00e-09 |
| coral1 | 331 | TM | 0.74 | 3.00e-09 |
| mediumorchid | 326 | TAT | -0.73 | 7.00e-09 |
| coral1 | 331 | TAT | 0.66 | 4.00e-07 |
| tan4 | 172 | Trig | 0.61 | 5.00e-06 |
| lightsteelblue | 132 | Folate | -0.61 | 6.00e-06 |
| ^a^ Statistical significance threshold p≤ 2.71e-09; suggestive threshold p≤6.25e-06  **Abbreviations**: PNS- previous number of strokes; F12- prothrombin fragments F1 + 2; TAT- thrombin-antithrombin complex; TM- thrombomodulin; Trig- plasma total triglycerides | | | | |
