# Supplementary material for: Multi-omic analysis of stroke recurrence in African Americans from the Vitamin Intervention for Stroke Prevention (VISP) clinical trial
Source: PLoS One. 2021 Mar 4;16(3):e0247257. doi: 10.1371/journal.pone.0247257 (PMC7932724; doi:10.1371/journal.pone.0247257)
Supplement: S7 Table — (DOCX) [file pone.0247257.s008.docx]

**S7 Table. Significant WGCNA module (DNA methylation)-trait (metabolite) associations.**

| **Module** | **# of Loci/Module** | **Correlated Metabolite** | **Sub Pathway** | **r** | **P^a^** |
| --- | --- | --- | --- | --- | --- |
| lightcyan | 1058 | benzoylecgonine | Drug - Analgesics, Anesthetics | 0.98 | 9.00e-32 |
| honeydew | 62 | sertraline | Drug - Psychoactive | 0.97 | 2.00e-29 |
| skyblue1 | 142 | escitalopram | Drug - Psychoactive | -0.9 | 3.00e-23 |
| plum4 | 36 | carbamazepine 10,11-epoxide | Drug - Neurological | 0.94 | 8.00e-23 |
| brown4 | 177 | 5-HETE | Eicosanoid | -0.9 | 2.00e-22 |
| darkolivegreen2 | 45 | escitalopram | Drug - Psychoactive | 0.93 | 3.00e-21 |
| darkolivegreen | 284 | threonylphenylalanine | Dipeptide | -0.9 | 5.00e-21 |
| firebrick4 | 125 | cetirizine | Drug - Respiratory | -0.9 | 1.00e-20 |
| thistle1 | 165 | carbamazepine 10,11-epoxide | Drug - Neurological | -0.9 | 2.00e-20 |
| lightpink3 | 77 | 5-HETE | Eicosanoid | 0.92 | 3.00e-20 |
| pink | 3174 | benzoylecgonine | Drug - Analgesics, Anesthetics | -0.9 | 6.00e-20 |
| navajowhite2 | 161 | sertraline | Drug - Psychoactive | -0.9 | 9.00e-19 |
| bisque4 | 176 | cetirizine | Drug - Respiratory | 0.91 | 2.00e-18 |
| lightsteelblue1 | 214 | threonylphenylalanine | Dipeptide | 0.89 | 8.00e-17 |
| navajowhite2 | 161 | desmethylnaproxen sulfate | Drug - Analgesics, Anesthetics | -0.8 | 7.00e-13 |
| indianred3 | 48 | lisinopril | Drug - Cardiovascular | 0.81 | 4.00e-12 |
| darkolivegreen | 284 | salicylic acid acyl-beta-D-glucuronide | Drug - Topical Agents | -0.8 | 6.00e-12 |
| yellow3 | 52 | enalapril | Drug - Cardiovascular | 0.81 | 8.00e-12 |
| honeydew | 62 | desmethylnaproxen sulfate | Drug - Analgesics, Anesthetics | 0.79 | 3.00e-11 |
| lightsteelblue1 | 214 | salicylic acid acyl-beta-D-glucuronide | Drug - Topical Agents | 0.79 | 5.00e-11 |
| mediumpurple3 | 219 | benzoylecgonine | Drug - Analgesics, Anesthetics | -0.8 | 5.00e-11 |
| navajowhite2 | 161 | naproxen | Drug - Analgesics, Anesthetics | -0.8 | 8.00e-11 |
| lightcyan1 | 192 | triamterene | Drug - Cardiovascular | -0.8 | 1.00e-10 |
| lightsteelblue | 132 | enalapril | Drug - Cardiovascular | -0.8 | 1.00e-10 |
| darkslateblue | 175 | lisinopril | Drug - Cardiovascular | -0.8 | 6.00e-10 |
| white | 365 | iminodiacetate (IDA) | Chemical | -0.8 | 1.00e-09 |
| honeydew | 62 | naproxen | Drug - Analgesics, Anesthetics | 0.74 | 3.00e-09 |
| lavenderblush2 | 73 | citrate | TCA Cycle | -0.7 | 3.00e-09 |
| lavenderblush3 | 157 | citrate | TCA Cycle | 0.74 | 3.00e-09 |
| darkgreen | 553 | iminodiacetate (IDA) | Chemical | 0.72 | 8.00e-09 |
| grey60 | 905 | cimetidine | Drug - Gastrointestinal | 0.72 | 1.00e-08 |
| lavenderblush3 | 157 | 2-hydroxyglutarate | Fatty Acid, Dicarboxylate | 0.72 | 1.00e-08 |
| coral3 | 53 | furosemide | Drug - Cardiovascular | 0.71 | 2.00e-08 |
| steelblue | 306 | p-cresol sulfate | Benzoate Metabolism | 0.68 | 1.00e-07 |
| darkolivegreen | 284 | theanine | Food Component/Plant | -0.70 | 2.00e-07 |
| lightcoral | 132 | threonylphenylalanine | Dipeptide | 0.67 | 2.00e-07 |
| lightsteelblue1 | 214 | theanine | Food Component/Plant | 0.67 | 2.00e-07 |
| navajowhite1 | 80 | thyroxine | Tyrosine Metabolism | -0.7 | 2.00e-07 |
| ^a^ Statistical significance threshold: p≤1.14e-10 (bold); suggestive threshold: p≤2.60e-07 | | | | | |
