# Supplementary material for: Multi-omic analysis of stroke recurrence in African Americans from the Vitamin Intervention for Stroke Prevention (VISP) clinical trial
Source: PLoS One. 2021 Mar 4;16(3):e0247257. doi: 10.1371/journal.pone.0247257 (PMC7932724; doi:10.1371/journal.pone.0247257)
Supplement: S8 Table — (DOCX) [file pone.0247257.s009.docx]

**S8 Table. WGCNA module (metabolite)-trait (DNA methylation loci) associations**.

| **Module** | **# of metabolites/ module** | **Primary Pathways** | **Correlated Locus** | **CHR:BP^a^** | **Nearest Gene** | **Locus Location** | **r** | **P^b^** |  |
| --- | --- | --- | --- | --- | --- | --- | --- | --- | --- |
| green | 52 | LPL; PC synthesis; plasmalogen, vitamin A metabolism | cg18461635 | 3:46990143 | *CCDC12* | Body | -0.65 | 2.00e-06 |  |
| turquoise | 214 | AA metabolism; xenobiotics; FA synthesis | cg19536075 | 8:143474651 | *TSNARE1* | 5'UTR | 0.63 | 4.00e-06 |  |
| black | 34 | Steroids Androgenic, pregeneolone, progestin; hemoglobin/  porphyrin metabolism | cg11516031 | 4:15776675 | *CD38* | Upstream 2.7 kb | -0.63 | 5.00e-06 |  |
| black | 34 | Steroids Androgenic, pregeneolone, progestin; hemoglobin/  porphyrin metabolism | cg18756557 | 12:1609369 | *LOC100292680* | TSS1500 | -0.62 | 6.00e-06 |  |
| turquoise | 214 | AA metabolism; xenobiotics; FA synthesis | cg10123662 | 1:17865713 | *ARHGEF10L* | TSS1500 | 0.62 | 7.00e-06 |  |
| green | 52 | LPL; PC synthesis; plasmalogen, vitamin A metabolism | cg16567172 | 7:75931606 | *HSPB1* | TSS1500 | -0.62 | 7.00e-06 |  |
| black | 34 | Steroids Androgenic, pregeneolone, progestin; hemoglobin/  porphyrin metabolism | cg01177907 | 16:3184694 | *ZNF213* | TSS1500 | -0.61 | 9.00e-06 |  |
| turquoise | 214 | AA metabolism; xenobiotics; FA synthesis | cg25281481 | 17:7155697 | *C17orf81;*  *DULLARD* | 5'UTR;  TSS1500 | -0.61 | 1.00e-05 |  |
| black | 34 | Steroids Androgenic, pregeneolone, progestin; hemoglobin/  porphyrin metabolism | cg12666827 | 17:81043176 | *METRNL* | Body | -0.6 | 1.00e-05 |  |
| brown | 77 | Ceramindes; dihydro-sphingomyelins; sphingomyelins; hexosylceramide; PC synthesis | cg12382902 | 12:4383699 | *CCND2* | Body | -0.61 | 1.00e-05 |  |
| turquoise | 214 | AA metabolism; xenobiotics; FA synthesis | cg12297590 | 6:158080750 | *ZDHHC14* | Body | 0.61 | 1.00e-05 |  |
| black | 34 | Steroids Androgenic, pregeneolone, progestin; hemoglobin/ porphyrin metabolism | cg12491966 | 21:46925242 | *COL18A1* | Body | -0.6 | 1.00e-05 |  |
| yellow | 55 | Benzoate metabolism; tobacco metabolites; xanthan metabolism | cg03486832 | 12:122019525 | *KDM2B* | TSS1500 | 0.6 | 1.00e-05 |  |
| black | 34 | Steroids Androgenic, pregeneolone, progestin; hemoglobin/ porphyrin metabolism | cg22995449 | 14:75988765 | *BATF* | TSS200 | -0.6 | 1.00e-05 |  |
| turquoise | 214 | AA metabolism; xenobiotics; FA synthesis | cg16065768 | 12:114107712 | *LHX5* | Upstream 197kb | 0.61 | 1.00e-05 |  |
| black | 34 | Steroids Androgenic, pregeneolone, progestin; hemoglobin/ porphyrin metabolism | cg25645687 | 3:138664505 | *FOXL2* | 1stExon | -0.59 | 2.00e-05 |  |
| brown | 77 | Ceramindes; dihydro-sphingomyelins; sphingomyelins; hexosylceramide; PC synthesis | cg12059147 | 1:43613627 | *FAM183A* | 1stExon | -0.6 | 2.00e-05 |  |
| black | 34 | Steroids Androgenic, pregeneolone, progestin; hemoglobin/ porphyrin metabolism | cg18233405 | 8:98290148 | *TSPYL5* | 1stExon | -0.59 | 2.00e-05 |  |
| black | 34 | Steroids Androgenic, pregeneolone, progestin; hemoglobin/ porphyrin metabolism | cg01166994 | 20:58515340 | *PPP1R3D;*  *C20orf177* | 1stExon;  TSS1500 | -0.6 | 2.00e-05 |  |
| black | 34 | Steroids Androgenic, pregeneolone, progestin; hemoglobin/ porphyrin metabolism | cg07682179 | 12:132400550 | *ULK1* | Body | -0.59 | 2.00e-05 |  |
| black | 34 | Steroids Androgenic, pregeneolone, progestin; hemoglobin/ porphyrin metabolism | cg12466950 | 5:1634049 | *LOC728613* | Body | -0.6 | 2.00e-05 |  |
| black | 34 | Steroids Androgenic, pregeneolone, progestin; hemoglobin/ porphyrin metabolism | cg11617484 | 10:135113459 | *TUBGCP2* | Body | -0.6 | 2.00e-05 |  |
| black | 34 | Steroids Androgenic, pregeneolone, progestin; hemoglobin/ porphyrin metabolism | cg12465412 | 1:233428450 | *PCNXL2* | Body | -0.6 | 2.00e-05 |  |
| turquoise | 214 | AA metabolism; xenobiotics; FA synthesis | cg13884376 | 9:130505349 | *SH2D3C* | Body | 0.59 | 2.00e-05 |  |
| black | 34 | Steroids Androgenic, pregeneolone, progestin; hemoglobin/ porphyrin metabolism | cg07571951 | 7:75931608 | *HSPB1* | TSS1500 | 0.6 | 2.00e-05 |  |
| black | 34 | Steroids Androgenic, pregeneolone, progestin; hemoglobin/ porphyrin metabolism | cg22046688 | 18:7122191 | *LAMA1* | Upstream 4.1kb | 0.6 | 2.00e-05 |  |
| black | 34 | Steroids Androgenic, pregeneolone, progestin; hemoglobin/ porphyrin metabolism | cg14092626 | 9:79629668 | *FOXB2* | Upstream 3.7kb | -0.6 | 2.00e-05 |  |
| turquoise | 214 | AA metabolism; xenobiotics; FA synthesis | cg13914486 | 10:6117324 | *IL2RA* | Upstream 11.7kb | 0.59 | 2.00e-05 |  |
| brown | 77 | Ceramindes; dihydro-sphingomyelins; sphingomyelins; hexosylceramide; PC synthesis | cg13343159 | 8:700290 | *ERICH1* | Upstream 18.2kb | 0.59 | 2.00e-05 |  |
| black | 34 | Steroids Androgenic, pregeneolone, progestin; hemoglobin/ porphyrin metabolism | cg02414710 | 1:232650142 | *SIPA1L2* | 1stExon | -0.58 | 3.00e-05 |  |
| black | 34 | Steroids Androgenic, pregeneolone, progestin; hemoglobin/ porphyrin metabolism | cg11008718 | 8:33370666 | *C8orf41* | 1stExon | -0.58 | 3.00e-05 |  |
| black | 34 | Steroids Androgenic, pregeneolone, progestin; hemoglobin/ porphyrin metabolism | cg12766967 | 10:75561895 | *NDST2* | 3'UTR | -0.59 | 3.00e-05 |  |
| grey | 337 | AA metabolism; xenobiotics | cg25377665 | 17:36903811 | *PCGF2* | 5'UTR | 0.59 | 3.00e-05 |  |
| black | 34 | Steroids Androgenic, pregeneolone, progestin; hemoglobin/ porphyrin metabolism | cg02877657 | 2:55493212 | *MTIF2* | 5'UTR | -0.59 | 3.00e-05 |  |
| black | 34 | Steroids Androgenic, pregeneolone, progestin; hemoglobin/ porphyrin metabolism | cg04042305 | 15:55562773 | *RAB27A* | 5'UTR | -0.58 | 3.00e-05 |  |
| turquoise | 214 | AA metabolism; xenobiotics; FA synthesis | cg11995069 | 11:47600522 | *KBTBD4;*  *NDUFS3* | 5'UTR;  TSS200 | -0.59 | 3.00e-05 |  |
| turquoise | 214 | AA metabolism; xenobiotics; FA synthesis | cg10698549 | 1:24969970 | *SRRM1* | Body | 0.58 | 3.00e-05 |  |
| black | 34 | Steroids Androgenic, pregeneolone, progestin; hemoglobin/ porphyrin metabolism | cg01726287 | 14:93812936 | *COX8C;*  *KIAA1409* | TSS1500;  5'UTR | -0.58 | 3.00e-05 |  |
| brown | 77 | Ceramindes; dihydro-sphingomyelins; sphingomyelins; hexosylceramide; PC synthesis | cg03150474 | 6:10695108 | *C6orf52;*  *PAK1IP1* | TSS1500;  TSS200 | 0.59 | 3.00e-05 |  |
| black | 34 | Steroids Androgenic, pregeneolone, progestin; hemoglobin/ porphyrin metabolism | cg08754654 | 5:154026448 | *LARP1* | Upstream 64.5kb | -0.58 | 3.00e-05 |  |
| black | 34 | Steroids Androgenic, pregeneolone, progestin; hemoglobin/ porphyrin metabolism | cg10700019 | 8:145984882 | *ZNF251* | Upstream 2.9kb | -0.59 | 3.00e-05 |  |
| turquoise | 214 | AA metabolism; xenobiotics; FA synthesis | cg24144440 | 1:119526882 | *TBX15* | 5'UTR | 0.58 | 4.00e-05 |  |
| green | 52 | LPL; PC synthesis; plasmalogen, vitamin A metabolism | cg20678800 | 1:115857431 | *NGF* | 5'UTR | -0.58 | 4.00e-05 |  |
| black | 34 | Steroids Androgenic, pregeneolone, progestin; hemoglobin/ porphyrin metabolism | cg26224077 | 6:170860440 | *PSMB1* | Body | 0.58 | 4.00e-05 |  |
| turquoise | 214 | AA metabolism; xenobiotics; FA synthesis | cg15904834 | 7:11676110 | *THSD7A* | Body | 0.58 | 4.00e-05 |  |
| turquoise | 214 | AA metabolism; xenobiotics; FA synthesis | cg13696351 | 9:140063617 | *LRRC26* | Body | -0.58 | 4.00e-05 |  |
| turquoise | 214 | AA metabolism; xenobiotics; FA synthesis | cg11091004 | 6:29716804 | *LOC285830* | Body | 0.57 | 4.00e-05 |  |
| black | 34 | Steroids Androgenic, pregeneolone, progestin; hemoglobin/ porphyrin metabolism | cg01999046 | 9:139779708 | *TRAF2* | TSS1500 | -0.58 | 4.00e-05 |  |
| grey | 337 | AA metabolism; xenobiotics | cg26008916 | 17:8151220 | *PFAS;*  *C17orf68* | TSS1500;  Body | -0.57 | 4.00e-05 |  |
| black | 34 | Steroids Androgenic, pregeneolone, progestin; hemoglobin/ porphyrin metabolism | cg00494710 | 2:27633183 | *PPM1G* | TSS1500 | -0.58 | 4.00e-05 |  |
| turquoise | 214 | AA metabolism; xenobiotics; FA synthesis | cg18744021 | 19:56739788 | *ZSCAN5A* | TSS200 | 0.58 | 4.00e-05 |  |
| green | 52 | LPL; PC synthesis; plasmalogen, vitamin A metabolism | cg18073380 | 3:101396084 | *ZBTB11;*  *LOC100009676* | TSS200;  Body | -0.58 | 4.00e-05 |  |
| black | 34 | Steroids Androgenic, pregeneolone, progestin; hemoglobin/ porphyrin metabolism | cg12063412 | 19:18528152 | *SSBP4* | Upstream 969bp | -0.58 | 4.00e-05 |  |
| black | 34 | Steroids Androgenic, pregeneolone, progestin; hemoglobin/ porphyrin metabolism | cg24606762 | 20:61806972 | *MIR124-3* | Upstream 1.7kb | -0.58 | 4.00e-05 |  |
| turquoise | 214 | AA metabolism; xenobiotics; FA synthesis | cg15013761 | 17:25982249 | *LGALS9* | Downstream 6.1kb | 0.58 | 4.00e-05 |  |
| turquoise | 214 | AA metabolism; xenobiotics; FA synthesis | cg07190917 | 16:31045100 | *STX4* | 1stExon | 0.57 | 5.00e-05 |  |
| turquoise | 214 | AA metabolism; xenobiotics; FA synthesis | cg01240047 | 14:91224692 | *TTC7B* | Body | 0.57 | 5.00e-05 |  |
| turquoise | 214 | AA metabolism; xenobiotics; FA synthesis | cg18744792 | 10:126688687 | *CTBP2* | Body | 0.57 | 5.00e-05 |  |
| black | 34 | Steroids Androgenic, pregeneolone, progestin; hemoglobin/ porphyrin metabolism | cg16114706 | 22:46509464 | *LOC400931;*  *MIRLET7B* | Body;TSS200 | -0.57 | 5.00e-05 |  |
| black | 34 | Steroids Androgenic, pregeneolone, progestin; hemoglobin/ porphyrin metabolism | cg12237140 | 19:47988486 | *KPTN* | TSS1500 | -0.57 | 5.00e-05 |  |
| black | 34 | Steroids Androgenic, pregeneolone, progestin; hemoglobin/ porphyrin metabolism | cg17607231 | 2:231090329 | *SP140* | TSS200 | -0.57 | 5.00e-05 |  |
| turquoise | 214 | AA metabolism; xenobiotics; FA synthesis | cg13394713 | 8:79031638 | *PKIA* | Upstream 396kb | 0.57 | 5.00e-05 |  |
| brown | 77 | Ceramindes; dihydro-sphingomyelins; sphingomyelins; hexosylceramides; PC synthesis | cg21977766 | 11:1107580 | *MUC2* | Downstream 2.4kb | 0.57 | 5.00e-05 |  |
| brown | 77 | Ceramindes; dihydro-sphingomyelins; sphingomyelins; hexosylceramide; PC synthesis | cg14111685 | 2:242930205 | *LOC728323* | Upstream 99.3kb | 0.57 | 5.00e-05 |  |
| ^a^ Base position based on hg19 ^b^ Statistical significance threshold p≤1.14e-10; suggestive threshold p≤5.42e-05  **Abbreviations**: LPL- lysophospholipids; PC- phosphotidylcholine; AA- amino acid; FA- fatty acid; UTR- untranslated region; TSS- transcriptional start site; kb- kilobase pairs | | | | | | | | | |
