# Supplementary material for: Multi-omic analysis of stroke recurrence in African Americans from the Vitamin Intervention for Stroke Prevention (VISP) clinical trial
Source: PLoS One. 2021 Mar 4;16(3):e0247257. doi: 10.1371/journal.pone.0247257 (PMC7932724; doi:10.1371/journal.pone.0247257)
Supplement: S9 Table — (DOCX) [file pone.0247257.s010.docx]

**S9 Table. Summary of significant metabolites-methylation associations using fully adjusted regression models.**

| **Metabolites** | **Pathway** | **Number of suggestive loci** | **Most significant locus per metabolite** | | | | | |
| --- | --- | --- | --- | --- | --- | --- | --- | --- |
|  |  |  | **Locus** | **Chr** | **BP^a^** | **Gene** | **Locus Location** | **P^b^** |
| 2-naphthol sulfate | Xenobiotics: Chemical | 245 | cg12743978 | 21 | 19617145 | *CHODL* | TSS200 | 4.40e-11 |
| (2,4 or 2,5) dimethylphenol sulfate | Xenobiotics: Food Component/Plant | 2023 | cg18801806 | 11 | 118869385 | *CCDC84* | Body- Intron | 5.19e-10 |
| o-cresol-sulfate | Xenobiotics: Benzoate Metabolism | 192 | cg07908160 | 17 | 56296982 | *MKS1* | TSS200 | 8.45e-10 |
| 2-ethylphenyl sulfate | Xenobiotics: Benzoate Metabolism | 130 | cg21733363 | 11 | 62623427 | *SLC3A2* | TSS1500 | 7.68e-09 |
| cotinine | Xenobiotics: Tobacco Metabolite | 31 | cg15574437 | 7 | 2019874 | *MAD1L1* | Body- Intron | 2.02e-08 |
| hydroxycotinine | Xenobiotics: Tobacco Metabolite | 1 | cg24141863 | 4 | 176986845 | *WDR17* | TSS200 | 6.51e-08 |
| ^a^ Base position based on hg19 ^b^ Statistical significance threshold: p≤1.14e-10; suggestive threshold: p≤1.05e-07 | | | | | | | | |
