# Supplementary material for: Multi-omic analysis of stroke recurrence in African Americans from the Vitamin Intervention for Stroke Prevention (VISP) clinical trial
Source: PLoS One. 2021 Mar 4;16(3):e0247257. doi: 10.1371/journal.pone.0247257 (PMC7932724; doi:10.1371/journal.pone.0247257)
Supplement: Attachment — Submitted filename: Response to Reviewers .docx [file pone.0247257.s011.docx]

The authors express their appreciation and gratitude to the editor and reviewers for their thoughtful critiques. Below, please find our itemized responses which correspond to **bold underlined text** and corresponding tracked changes throughout the manuscript.

**From Editor:**

When submitting your revision, we need you to address these additional requirements.

1. Please ensure that your manuscript meets PLOS ONE's style requirements, including those for file naming. The PLOS ONE style templates can be found at

<https://journals.plos.org/plosone/s/file?id=wjVg/PLOSOne_formatting_sample_main_body.pdf> and

<https://journals.plos.org/plosone/s/file?id=ba62/PLOSOne_formatting_sample_title_authors_affiliations.pdf>

**Response:** We have formatted our documents to meet PLOS ONE’s style requirements.

2. We note that you have stated that you will provide repository information for your data at acceptance. Should your manuscript be accepted for publication, we will hold it until you provide the relevant accession numbers or DOIs necessary to access your data. If you wish to make changes to your Data Availability statement, please describe these changes in your cover letter and we will update your Data Availability statement to reflect the information you provide.

**Response:** We have modified the text to include the following: The Genomics and Randomized Trials Network (GARNET) Vitamin Intervention Stroke Prevention (VISP) trial data is available in the database of Genotypes and Phenotypes (dbGaP)- dbGaP Study Accession: phs000343.v3.p1.

**Comments to the Author:**

1. Is the manuscript technically sound, and do the data support the conclusions?

The manuscript must describe a technically sound piece of scientific research with data that supports the conclusions. Experiments must have been conducted rigorously, with appropriate controls, replication, and sample sizes. The conclusions must be drawn appropriately based on the data presented.

Reviewer #1: Yes

Reviewer #2: Yes

2. Has the statistical analysis been performed appropriately and rigorously?

Reviewer #1: Yes

Reviewer #2: Yes

3. Have the authors made all data underlying the findings in their manuscript fully available?

The [PLOS Data policy](https://nam02.safelinks.protection.outlook.com/?url=http%3A%2F%2Fwww.plosone.org%2Fstatic%2Fpolicies.action%23sharing&data=04%7C01%7Ckeenek%40ecu.edu%7Cd1c8848d271d4d6aa29d08d8a857589b%7C17143cbb385c4c45a36ac65b72e3eae8%7C0%7C0%7C637444440403421965%7CUnknown%7CTWFpbGZsb3d8eyJWIjoiMC4wLjAwMDAiLCJQIjoiV2luMzIiLCJBTiI6Ik1haWwiLCJXVCI6Mn0%3D%7C1000&sdata=58IakcA9yjss34B%2BDFFDlsYkmszu421Vnz9i5iYMLRE%3D&reserved=0) requires authors to make all data underlying the findings described in their manuscript fully available without restriction, with rare exception (please refer to the Data Availability Statement in the manuscript PDF file). The data should be provided as part of the manuscript or its supporting information, or deposited to a public repository. For example, in addition to summary statistics, the data points behind means, medians and variance measures should be available. If there are restrictions on publicly sharing data—e.g. participant privacy or use of data from a third party—those must be specified.

Reviewer #1: Yes

Reviewer #2: Yes

4. Is the manuscript presented in an intelligible fashion and written in standard English?

PLOS ONE does not copyedit accepted manuscripts, so the language in submitted articles must be clear, correct, and unambiguous. Any typographical or grammatical errors should be corrected at revision, so please note any specific errors here.

Reviewer #1: Yes

Reviewer #2: Yes

5. Review Comments to the Author

Please use the space provided to explain your answers to the questions above. You may also include additional comments for the author, including concerns about dual publication, research ethics, or publication ethics. (Please upload your review as an attachment if it exceeds 20,000 characters)

Reviewer #1: This study suggests results that are helpful in finding biomarkers related to stroke recurrence in high-risk groups.

To further improve this research method, it would be better to describe the type of ischemic stroke or the lesion of the stroke in the subjects of this study. The relationship between these and biomarkers could lead to better research results.

If this is not possible, it would be better to present it as a further study in the discussion.

Reviewer #2: This manuscript is showing characteristic candidates of stroke based on metabolomics, genetic variations and epigenomics. statisitc analysis was carefully performed and the results showed interesting insights. Therefore, it is very excellent and acceptable for this journal.

**Response:** Based on the inclusion and exclusion criteria for the VISP trial, the current study focuses on nondisabling ischemic stroke. The study specifically excluded those with stroke due to cardioembolic source including atrial fibrillation, arterial dissection, and moderate to severe carotid stenosis considered for surgical revascularization. Although ischemic stroke subtyping is not available for participants in VISP, the study population is comprised of patients with stroke primarily due to small vessel occlusion or intracranial large artery atherosclerosis with some individuals with non-surgical extracranial large artery atherosclerosis and cryptogenic/ undetermined stroke subtypes. We have modified the methods section by adding the inclusion/exclusion criteria and modified the discussion to reflect our statement above.

**Edits to Methods-** VISP inclusion/exclusion criteria are listed below (8).

Inclusion criteria included:

1. Participants aged 35 years or older with elevated baseline homocysteine levels at or above the 25th percentile;
2. Participant enrollment within 120 days of suffering a non-disabling cerebral infarction characterized by the sudden onset of a neurological deficit persisting at least 24 hours and observed on CT or MRI;
3. Geographical accessibility for follow-up;
4. Adequate means of transportation;
5. Compliance of 75% or greater with vitamin regiment in the run-in period and
6. Patient agreement to take study medication and avoid other vitamin supplements containing folic acid and B6

VISP exclusion criteria included:

1. Stroke due to any form of intracranial hemorrhage, dissection of a cervico-cephalic artery, veno-occlusive disease, drug abuse, or vasculitis;
2. CT or MRI of brain showing lesion other than ischemic infarction as cause;
3. Modified Rankin Stroke Scale (RSS) score of 4-5 at time of eligibility determination;
4. Presence of specific potential sources of cardiogenic emboli, such as atrial fibrillation within 30 days of stroke or history of prosthetic cardiac valve, intracardiac thrombus or neoplasm, or valvular vegetation;
5. Presence of major neurologic illness apart from stroke that would prevent proper evaluation of recurrent stroke;
6. Presence of cancer, pulmonary disease, or other illness which, in the opinion of the study physician, would limit the life expectancy of the patient to less than two years;
7. Severe congestive heart failure;
8. Renal insufficiency requiring dialysis;
9. Untreated pernicious anemia or untreated B12 deficiency;
10. Uncontrolled hypertension defined as systolic blood pressure >185 mm or diastolic >105 mm on two readings separated by five minutes at time of eligibility determination;
11. Conditions that prevent reliable participation in the study, such as refractory depression, severe cognitive impairment, alcoholism, or other substance abuse;
12. Use of medications, within the last 30 days, that affect homocysteine, such as methotrexate, tamoxifen, L-dopa, or phenytoin or bile acid sequestrants that can decrease folate levels
13. Woman of childbearing potential, defined as not having reached natural or surgical menopause or having had tubal ligation;
14. Participation in another trial in which active intervention is being received;
15. Participants on multivitamin supplements or single vitamins of B6 or folic acid were excluded unless they were willing to take the study supplements in place of the one(s) they usually took and/or

Any surgical procedure requiring a general anesthesia or hospital stay of three days or more, any type of invasive cardiac instrumentation, or an endarterectomy, stent placement, thrombectomy, or any other endovascular treatment of an abnormal carotid artery performed within 30 days prior to randomization or scheduled to be performed within 30 days after randomization.

**Edits to Discussion**- Although stroke subtype is not adjudicated in VISP, these stroke cases most likely represent small vessel variety due to the inclusion/exclusion criteria and higher proportion of small vessel (lacunar) ischemic stroke typically overserved in AA [41].

6. PLOS authors have the option to publish the peer review history of their article ([what does this mean?](https://nam02.safelinks.protection.outlook.com/?url=https%3A%2F%2Fjournals.plos.org%2Fplosone%2Fs%2Feditorial-and-peer-review-process%23loc-peer-review-history&data=04%7C01%7Ckeenek%40ecu.edu%7Cd1c8848d271d4d6aa29d08d8a857589b%7C17143cbb385c4c45a36ac65b72e3eae8%7C0%7C0%7C637444440403431957%7CUnknown%7CTWFpbGZsb3d8eyJWIjoiMC4wLjAwMDAiLCJQIjoiV2luMzIiLCJBTiI6Ik1haWwiLCJXVCI6Mn0%3D%7C1000&sdata=1T3gWhHvaWOhblHN9722gcFL5iuH27HRcGv8LQRWwlY%3D&reserved=0)). If published, this will include your full peer review and any attached files.


If you choose “no”, your identity will remain anonymous but your review may still be made public.


**Do you want your identity to be public for this peer review?** For information about this choice, including consent withdrawal, please see our [Privacy Policy](https://nam02.safelinks.protection.outlook.com/?url=https%3A%2F%2Fwww.plos.org%2Fprivacy-policy&data=04%7C01%7Ckeenek%40ecu.edu%7Cd1c8848d271d4d6aa29d08d8a857589b%7C17143cbb385c4c45a36ac65b72e3eae8%7C0%7C0%7C637444440403431957%7CUnknown%7CTWFpbGZsb3d8eyJWIjoiMC4wLjAwMDAiLCJQIjoiV2luMzIiLCJBTiI6Ik1haWwiLCJXVCI6Mn0%3D%7C1000&sdata=BbAhAjjejwHhBcHFeiWog0dMrAu4rqIsiVt0I5WEYcE%3D&reserved=0).

Reviewer #1: **Yes:** Seung-Bo Yang

Reviewer #2: **Yes:** Kazuo Ishii
